# Supplementary material for: The Cyclic Imine Core Common to the Marine Macrocyclic Toxins Is Sufficient to Dictate Nicotinic Acetylcholine Receptor Antagonism
Source: Mar Drugs. 2024 Mar 27;22(4):149. doi: 10.3390/md22040149 (PMC11050823; doi:10.3390/md22040149)
Supplement: Supplementary file 1 [file marinedrugs-22-00149-s001.zip › marinedrugs-2894029 supplementary materials-PM.pdf]

marine drugs

Supplementary Material

The Cyclic Imine Core Common to the Marine Macrocyclic Toxins Is Sufficient to Dictate Nicotinic Acetylcholine Receptor Antagonism

Yves Bourne [1], Gerlind Sulzenbacher [1], Laurent Chabaud [2,†], Rómulo Aráoz [3], Zoran Radić [4], Sandrine Conrod [5,‡], Palmer Taylor [4], Catherine Guillou [2], Jordi Molgó [3], Pascale Marchot [1,5,*]

[1] Lab "Architecture et Fonction des Macromolécules Biologiques" (AFMB), Aix-Marseille Univ, CNRS, Faculté des Sciences Campus Luminy, 13288 Marseille cedex 09, France; yves.bourne@univ-amu.fr (Y.B.); gerlind.sulzenbacher@univ-amu.fr (G.S.)
[2] Institut de Chimie des Substances Naturelles (ICSN), Univ Paris-Saclay, CNRS, 91198 Gif-sur-Yvette, France; catherine.guillou@cnrs.fr
[3] Service d'Ingénierie Moléculaire pour la Santé (SIMoS) EMR CNRS 9004, Département Médicaments et Technologies pour la Santé, Institut des Sciences du Vivant Frédéric Joliot, CEA, INRAE, Université Paris-Saclay, 91191 Gif-sur-Yvette, France; romulo.araoz@cea.fr (R.A.); jordi.molgo@cea.fr (J.M.)
[4] Skaggs School of Pharmacy and Pharmaceutical Sciences (SSPPS), University of California San Diego, La Jolla, CA, 92093-0751, USA; zradic@health.ucsd.edu (Z.R.); pwtaylor@health.ucsd.edu (P.T.)
[5] Centre de Recherche en Neurobiologie et Neurophysiologie de Marseille (CRN2M), Aix Marseille Univ, CNRS, 13344 Marseille, France
† Current address: Institut des Sciences Moléculaires (ISM), Univ Bordeaux, CNRS, Bordeaux INP, 33400 Talence, France; laurent.chabaud@u-bordeaux.fr
‡ Current address: CEREGE, Aix-Marseille Univ, CNRS, IRD, Collège de France, INRAE, 13545 Aix-en-Provence, France; conrod@cerege.fr
* Correspondence: pascale.marchot@univ-amu.fr

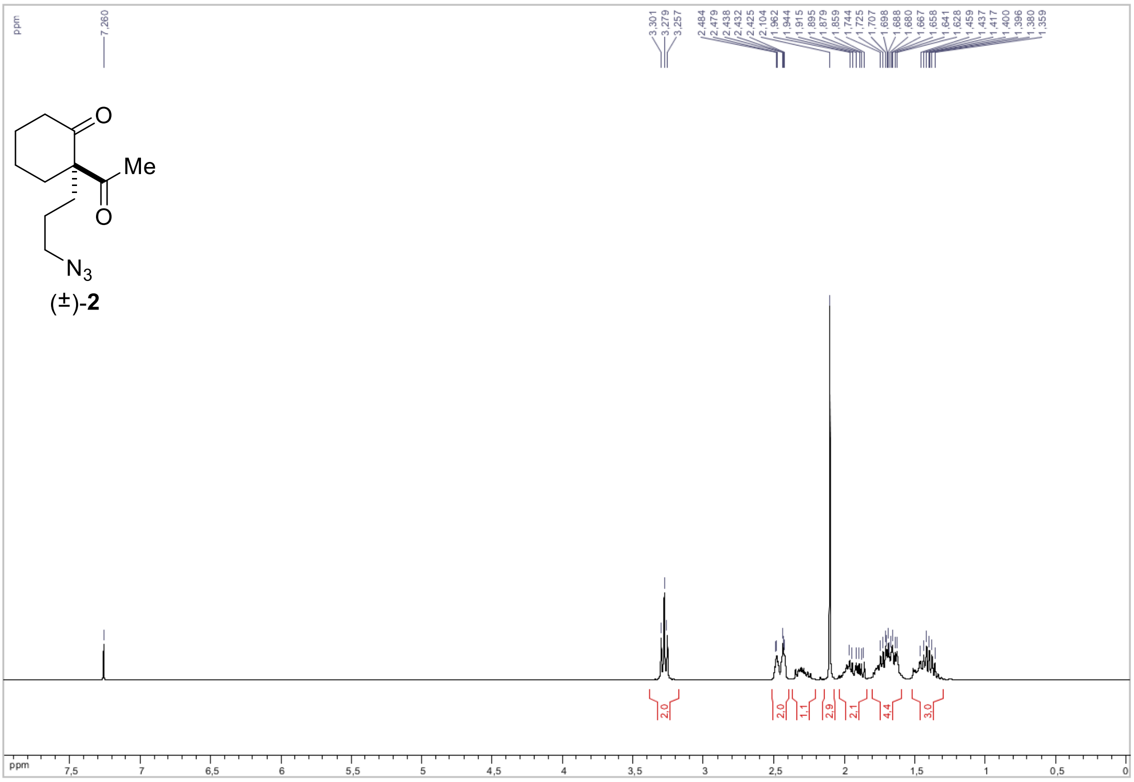

Figure S1. ^{1}H NMR spectrum ($CDCl_3$) of 2-acetyl-2-(3-azidopropyl)cyclohexanone (±)-**2**.

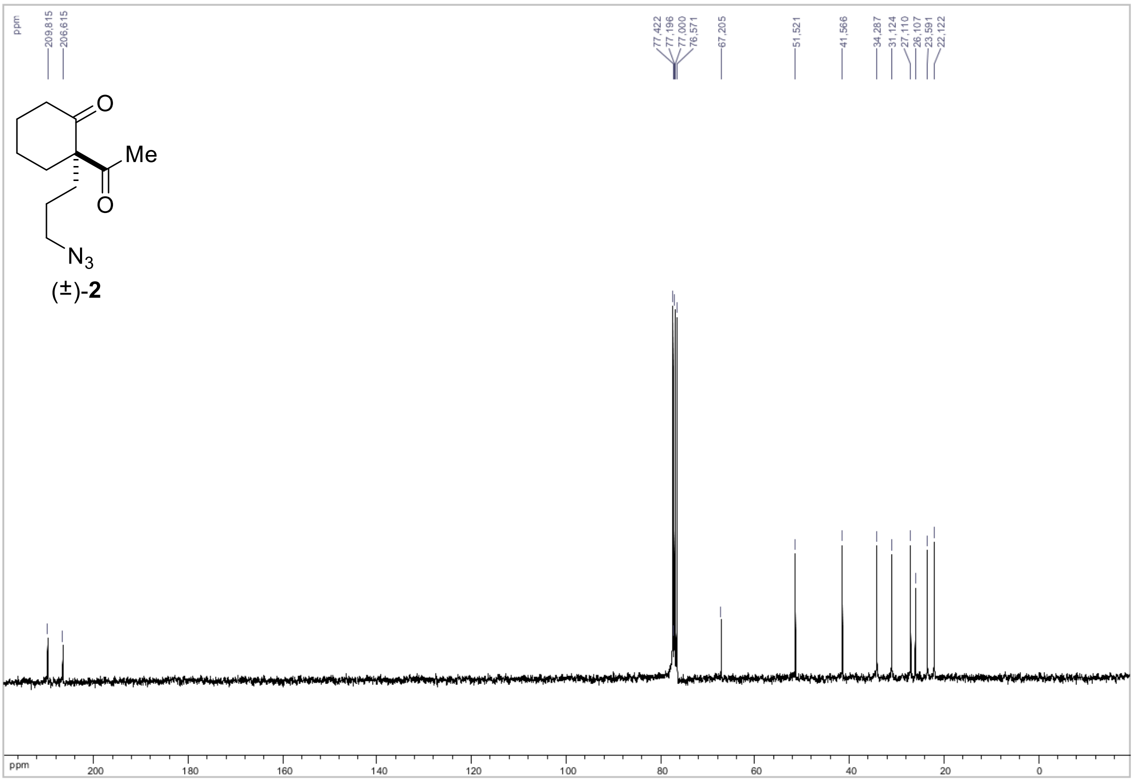

Figure S2. ^{13}C NMR spectrum ($CDCl_3$) of 2-acetyl-2-(3-azidopropyl)cyclohexanone (±)-**2**.

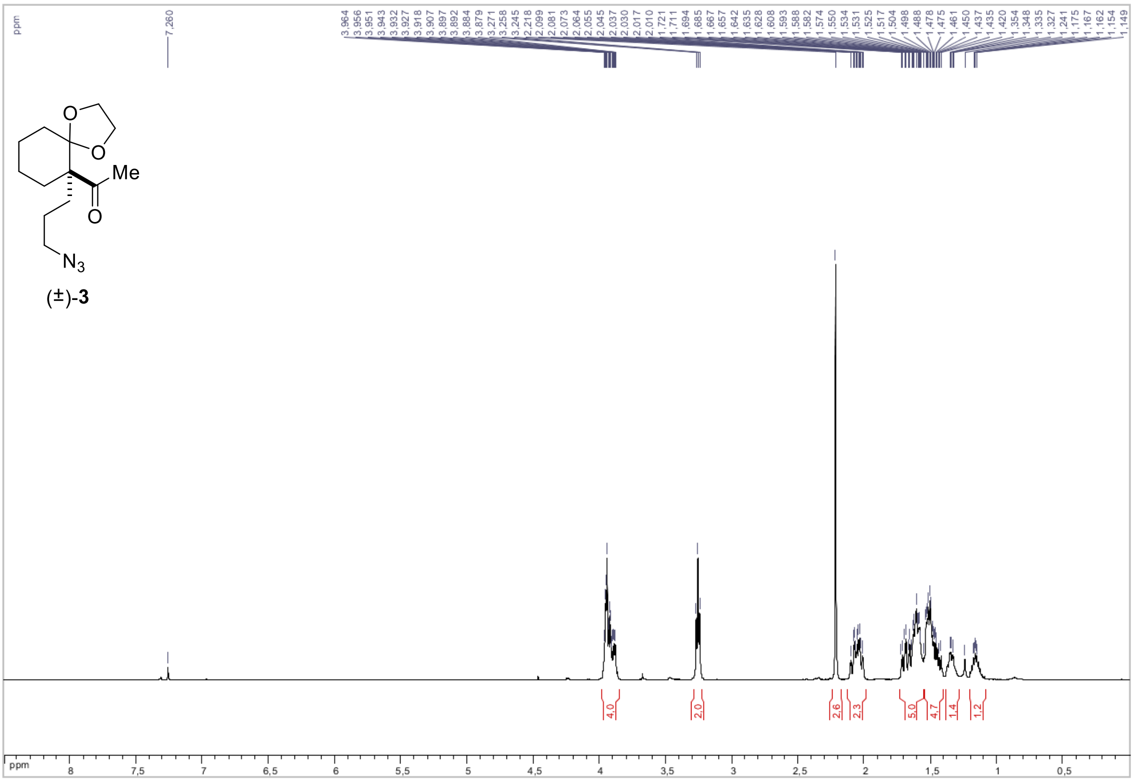

Figure S3. ^{1}H NMR spectrum ($CDCl_3$) of 1-[6-(3-azidopropyl)-1,4-dioxaspiro[4.5]dec-6-yl]ethanone (±)-**3**.

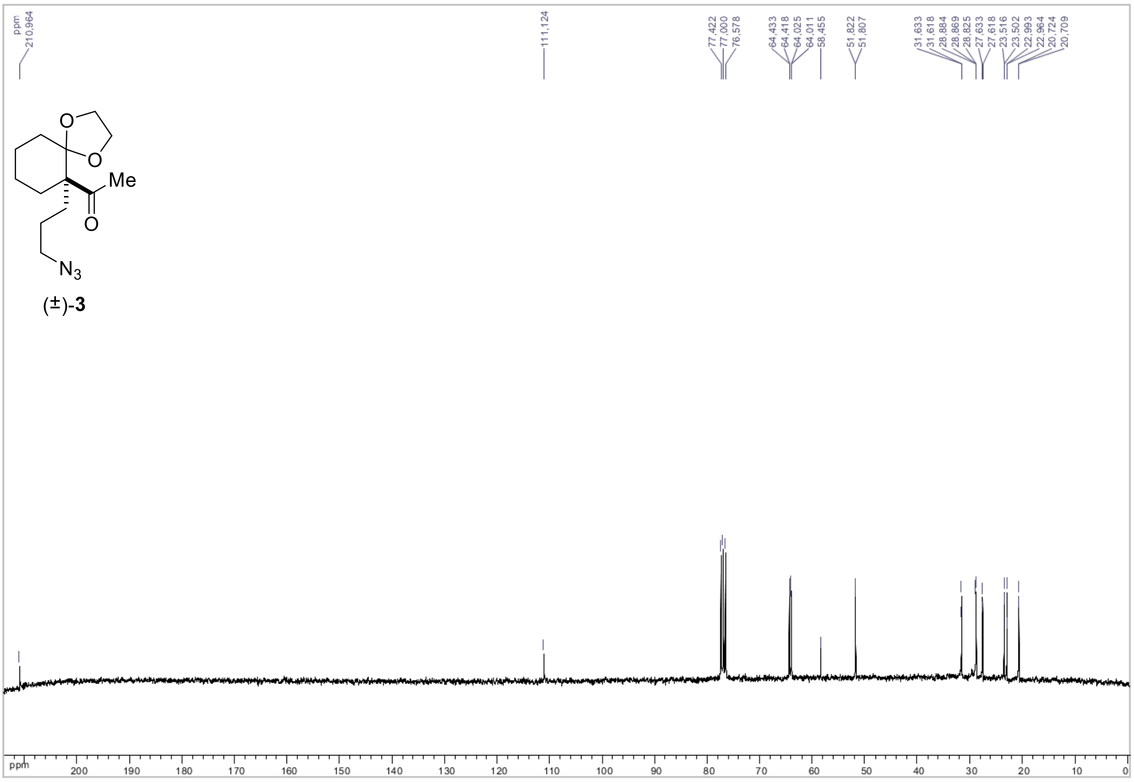

Figure S4. ^{13}C NMR spectrum ($CDCl_3$) of 1-[6-(3-azidopropyl)-1,4-dioxaspiro[4.5]dec-6-yl]ethanone (±)-**3**.

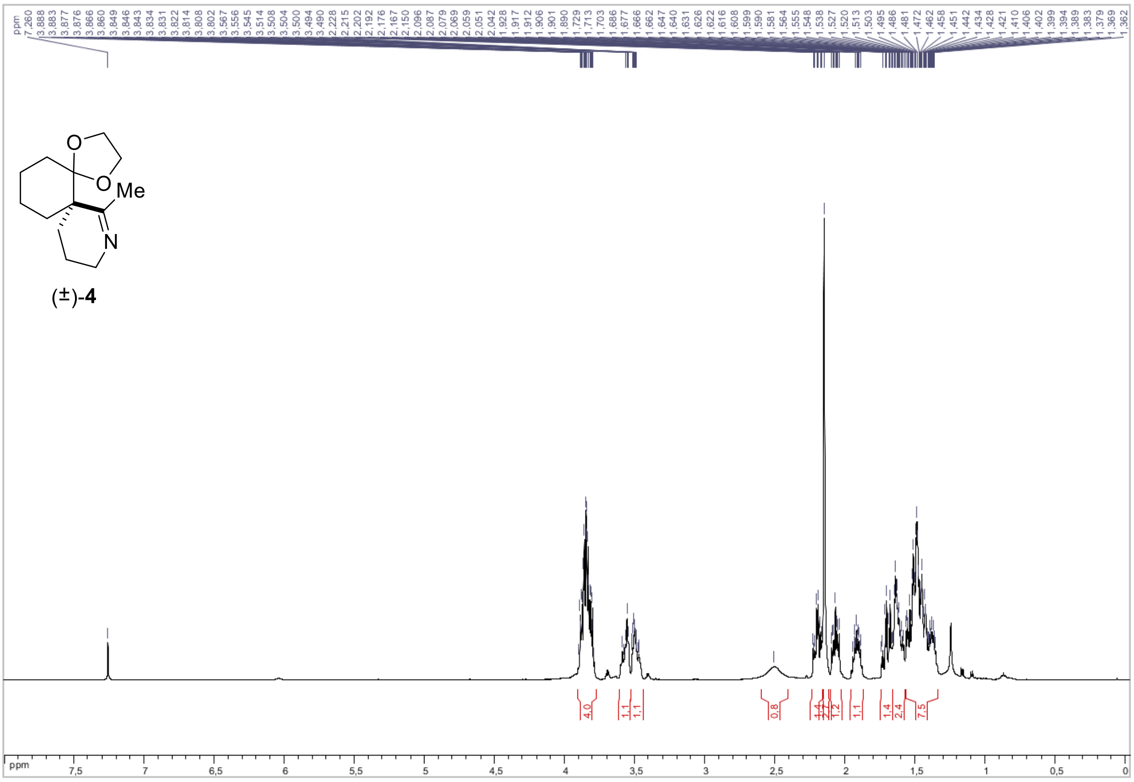

Figure S5. ^{1}H NMR spectrum ($CDCl_3$) of 7-methyl-1,4-dioxa-8-azadispiro[4.0.5.4]pentadec-7-ene (±)-**4**.

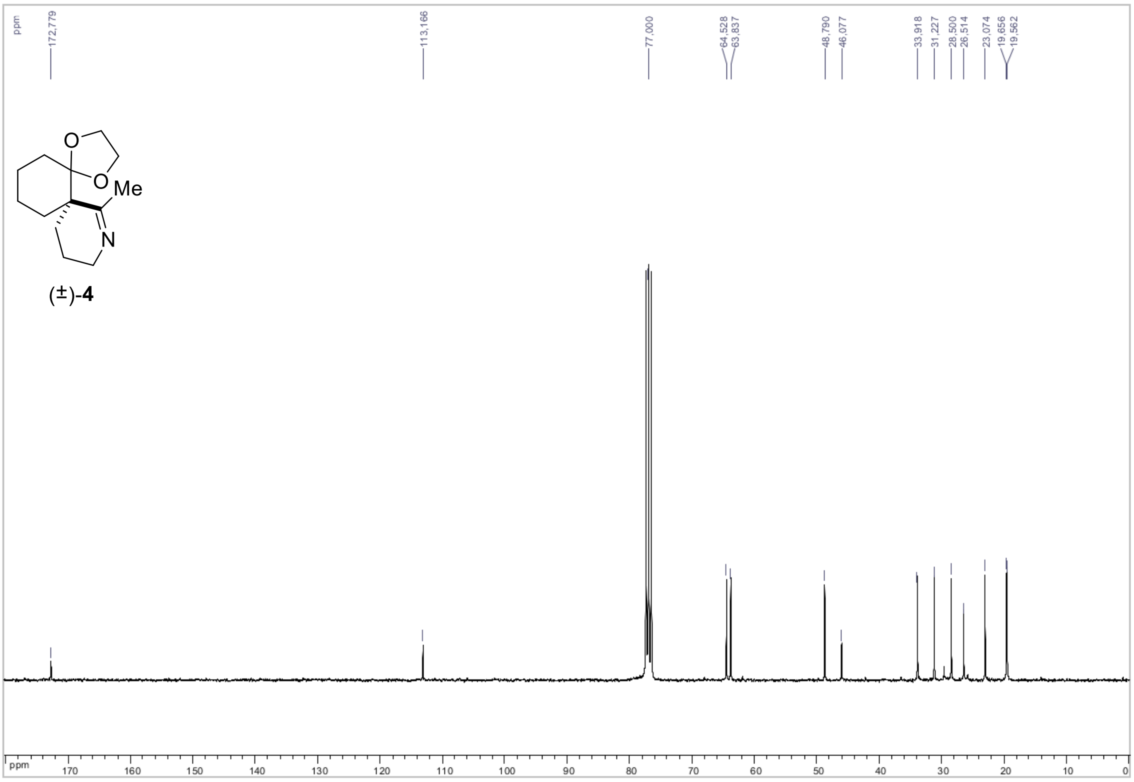

Figure S6. ^{13}C NMR spectrum ($CDCl_3$) of 7-methyl-1,4-dioxa-8-azadispiro[4.0.5.4]pentadec-7-ene (±)-**4**.

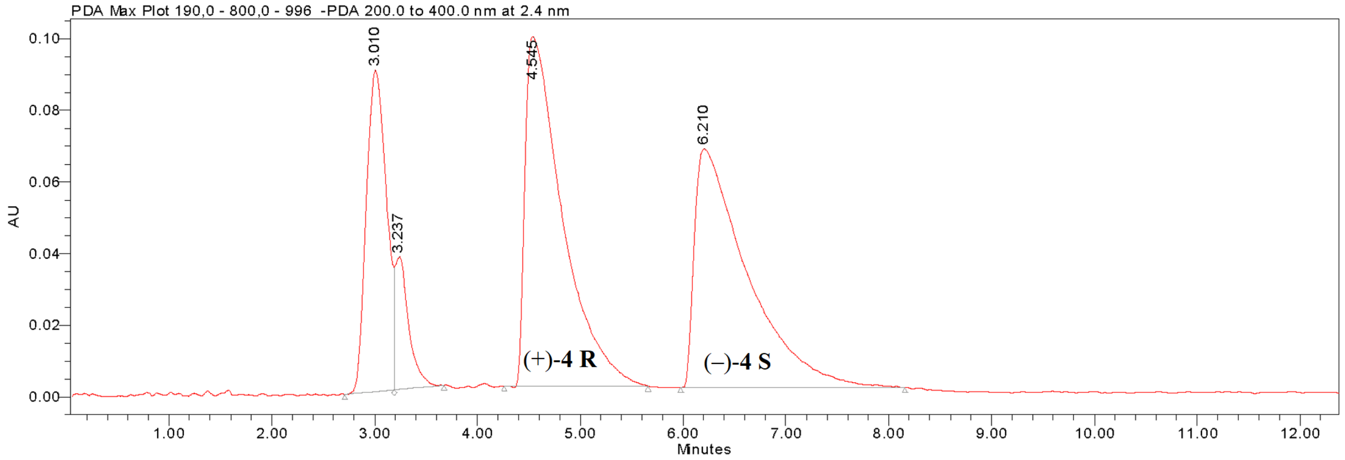

Figure S7. Separation of enantiomers (+)-**4** and (–)-**4** from the (±)-**4** racemate using chiral HPLC (Chiralcel® OD 10 µm 4.6x250 mm column, Hept/EtOH 80:20 (v/v), flow 1 mL/min).

Table S1. X-ray crystallography - Data collection and refinement statistics.

	Spiroimine (+)-**4 R**	Spiroimine (–)-**4 S**	Racemic (±)-**4**
Beamline	ESRF ID14-EH1	SOLEIL Proxima-1	ESRF ID23-1
**Data collection**			
Space group	$P2_12_12_1$	$P2_12_12_1$	$P2_12_12_1$
Cell dimensions *a*, *b*, *c* (Å)	87.98, 115, 129.96	88.34, 116.03, 131.04	75.79, 123.63, 131.1
Resolution [a] (Å)	48.13-2.00 (2.04-2.00)	48.49-1.85 (1.88-1.85)	65-2.10 (2.15-2.10)
No. reflections	89606 (4509)	109510 (5565)	72272 (4374)
CC (1/2)	0.999 (0.699)	1.0 (0.618)	0.993 (0.687)
R_{meas} [b]	0.087 (0.959)	0.050 (0.954)	0.128 (0.720)
$I / \sigma I$	15.5 (1.8)	22.2 (1.7)	8.7 (2.1)
Completeness (%)	100 (99.9)	94.9 (98.3)	99.7 (100)
Redundancy	6.2 (6.2)	7.0 (4.4)	3.5 (2.6)
Wilson B (A^2)	23.85	32.22	21.85
**Refinement**			
Resolution (Å)	48.18-2.00 (2.052-2.00)	47.97-1.85 (1.88-1.85)	65-2.1 (2.15-2.1)
No. reflections working set	84991 (6197)	103900 (7900)	70046 (5108)
No. reflections test set	4537 (317)	5509 (434)	2180 (148)
R_{work} / R_{free} [c]	0.1712/0.2060	0.1820/0.2114	0.18/0.21
No. atoms			
Protein + N-glycans	8457	8440	8560
Ligands/Ions	85	90	80
Water	784	706	520
B-factors			
Protein + N-glycans, main/side	32.92/38.29	38.67/43.39	33.7/38.5
Ligands/Ions	40	66.83	-
Water	37.38	42.06	36.4
R.m.s. deviations [d]			
Bond lengths (Å)	0.008	0.008	0.007
Bond angles (°)	1.472	1.447	1.271
Ramachandran			
Favoured (%)	98.94	99.04	98.5
Allowed (%)	1.06	0.96	1.5
Disallowed (%)	0	0	0
PDB accession code	*8Q1M*	*8QTL*	*8QX2*

[a] Values in parentheses are those for the highest-resolution shell. [b] $R_{meas} = \Sigma_h\Sigma_i(n_h/(n_h - 1))^{1/2}|I_{hi} - \langle I_h\rangle|/\Sigma_h\Sigma_i\langle I_h\rangle$, where I is an individual reflection measurement and <I> is the mean intensity for symmetry-related reflections. [c] $R_{work} = \Sigma_{hkl}||Fo(hkl)| - |Fc||/\Sigma_{hkl}|Fo(hkl)|$, where Fo and Fc are observed and calculated structure factors, respectively. R_{free} is calculated for 5% of randomly selected reflections excluded from refinement. [d] Root-mean-square deviation from ideal values.

Table S2. Geometric parameters of key interactions between the (+)-**4 R** and (–)-**4 S** enantiomers and side chains in the A-AChBP binding pocket.

Subunit interface	Spiroimine (+)-4 **R**			Spiroimine (-)-4 **S**		
	Type of interaction			Type of interaction		
	H-bonds*	π-π stacking	van der Waals**	H-bonds*	π-π stacking	van der Waals**
A-B	N3-W147 carbonyl oxygen 2.6 Å / 140.7°	Cyclic imine-W147 face-to-face	Y93, Y188, C190, Y195 *Y55, I118, S167*	N3-W147 carbonyl oxygen 2.9 Å / 123.8°	Cyclic imine-W147 T-shaped	Y93, Y188, C190, C191, Y195 *Y55, I118*
B-C	N3-W147 carbonyl oxygen 2.7 Å / 144.2°	Cyclic imine-W147 face-to-face	Y93, Y188, C190, Y195 *Y55, I118*	N3-W147 carbonyl oxygen 3.5 Å / 121.2°	Cyclic imine-W147 T-shaped	Y93, Y188, C190, C191, Y195 *Y55, I118*
C-D	N3-W147 carbonyl oxygen 2.7 Å / 142°	Cyclic imine-W147 face-to-face	Y93, Y188, C190, Y195 *Y55, I118*	N3-W147 carbonyl oxygen 2.8 Å / 125.3°	Cyclic imine-W147 T-shaped	Y93, Y188, C190, C191, Y195 *Y55, I118*
D-E	N3-W147 carbonyl oxygen 2.7 Å / 144.4°	Cyclic imine-W147 face-to-face	Y93, Y188, C190, Y195 *Y55, I118*	N3-W147 carbonyl oxygen 3.0 Å / 126.9°	Cyclic imine-W147 T-shaped	Y93, Y188, C190, C191, Y195 *Y55, I118*
E-A	N3-W147 carbonyl oxygen 2.7 Å / 142.1°	Cyclic imine-W147 face-to-face	Y93, Y188, C190, Y195 *Y55, I118*	N3-W147 carbonyl oxygen 2.9 Å / 125.6°	Cyclic imine-W147 T-shaped	Y93, Y188, C190, C191, Y195 *Y55, I118*

*The angle value refers to the $\hat{H}$ angle between the hydrogen bond donor, acceptor and acceptor antecedent.
**Non-italicized A-AChBP residues Y93, Y188, C190, C191, Y195 belong to the principal, (+) face of the subunit interface, while italicized residues *Y55, I118, S167* belong to the complementary, (-) face of the interface (Figure A1).

-- *End of Suppl. Mat.* -------------
